# Social Inequalities in Participation in Cervical Cancer Screening in a Metropolitan Area Implementing a Pilot Organised Screening Programme (Paris Region, France)

**DOI:** 10.3389/ijph.2022.1604562

**Published:** 2022-07-04

**Authors:** Céline Audiger, Thomas Bovagnet, Julia Bardes, Gaëlle Abihsera, Jérôme Nicolet, Michel Deghaye, Audrey Bochaton, Gwenn Menvielle

**Affiliations:** ^1^ Le Centre Régional de Coordination des Dépistages des Cancers-CRCDC, Paris, France; ^2^ Sorbonne Université, INSERM, Institut Pierre Louis d'épidémiologie et de Santé Publique (IPLESP UMRS), Paris, France; ^3^ Université Paris Nanterre, UMR CNRS 7533 LADYSS, Nanterre, France

**Keywords:** social inequalities and health inequalities, cervical cancer screening, organised programme, absolute and relative inequalities, Paris area

## Abstract

**Objectives:** We aimed to examine social inequalities in participation in cervical cancer screening (CCS) in a metropolitan area by implementing a pilot organised screening programme. The pilot programme consisted of sending invitations to women who did not perform a pap smear within the past 3 years, managing interventions to reach vulnerable women, training healthcare professionals, and organising follow-ups of abnormal pap smears.

**Methods:** We studied participation in CCS between January 2014 and December 2016 among 241,257 women aged 25–63 years old. To assess relative inequalities, Odds Ratios were computed using multilevel logistic regression. To assess absolute inequalities, the CCS coverage and the rate difference were calculated. Inequalities were computed by age and neighbourhood characteristics (social deprivation and proportion of single women).

**Results:** Disparities in participation in CCS were observed by age and social deprivation. For overall screening compared to opportunistic screening, disparities by age were larger (OR25-35_vs._55–64 = 2.13 [2.08–2.19] compared to 2.02 [1.96–2.07]), but disparities by social deprivation were decreased (OR10%_most_vs._10%_least_deprived = 2.09 [1.90–2.30] compared to 2.22 [2.02–2.44]).

**Conclusion:** Disparities in CCS participation remain despite the organised programme. To reduce these inequalities, free screening should be proposed and evaluated.

## Introduction

Cervical cancer is the fourth most commonly diagnosed cancer among women worldwide. In France, about 3000 new cases and 1000 deaths occurred in 2018 alone [1]. Cervical smears have proved to be effective in decreasing mortality and incidence [2, 3]; however, inequalities in participation in cervical cancer screening (CCS) have been consistently observed, with socially deprived, older, and single women less likely to be screened [4–7]. Fighting these inequalities is one of the main expected benefits of an organised cancer screening programme [8]. An organised programme can differ depending on the geographic area covered (for example, nationwide [9] or covering only specific regions [10, 11]), the targeted population (all the eligible women or only women who did not have a pap smear within the recommended interval [11]), the fees for the CCS (whether they are free of charge or a co-payment from the women is required [12]), and the mode of invitation (by the GP or by a letter of invitation [9]). Even though these situations can influence the magnitude of socioeconomic inequalities in CCS participation [8], there is no consensus in the literature on this subject regarding the effect of the implementation of an organised programme on inequalities in CCS participation [13, 14], with studies reporting no change [15], a decrease [8], or an increase in inequalities [16].

Until 2019 in France, the recommendations outlined that women aged 25–65 years should perform a cervical pap smear every 3 years. Uptaking a CCS was mainly an opportunistic decision. However, an organised pilot CCS programme was initiated in 2010 within 13 administrative geographical areas. Each step of the pilot programme was monitored. This pilot programme improved CCS coverage by 12 percentage points [17], but its impact on social inequalities in participation has not yet been assessed because no information on women’s socioeconomic position was available in the 13 areas. Geocoding of all women’s addresses has been performed in the Val de Marne (VDM), one of the areas located in the Paris region implementing the organised pilot CCS programme. This data enabled us to investigate social inequalities in participation in CCS in this metropolitan area.

Assessing social inequalities is challenging. If most studies estimate social inequalities on a relative scale, inequalities could also be computed using absolute measures. A relative measure of inequalities is useful for exploring statistical associations: it is expressed as a ratio of health outcomes between social groups and is independent of the level of the health outcome [18]. By contrast, an absolute measure of inequalities is expressed as a difference in health outcomes between social groups. It accounts for the initial level of the health outcome and assesses the social burden of the health outcome in the population. The need to use both absolute and relative measures is highlighted in the literature to accurately reflect the different dimensions of health inequalities [18, 19].

Using a large high-quality population-based sample that included CCS history from administrative records, we aimed to examine social inequalities in participation in CCS in a metropolitan area by implementing a pilot organised screening programme using both relative and absolute measures of inequalities.

## Methods

### Presentation of the Organised CCS Programme

Once every 3 months, a list of all 25–65-year-old female residents of the VDM and the date of their previous pap smear was sent to the VDM screening management centre in charge of implementing the organised CCS programme by the VDM health insurance fund (with additional information from labs). The VDM screening management centre then identifies women who had not been screened over the past 3 years and sends them a personal invitation for screening by post. Recipients (or their next-of-kin) can respond to the letter with the date of their most recent pap smear or any reason for non-participation (hysterectomy, history of cervical cancer, disability which rendered the test impossible, personal objection, death). Upon the reception of the letter of invitation, women have to book an appointment with a medical professional of their choice. To help them, the letter specifies the different healthcare providers performing cervical smear tests: gynaecologists (in France they perform 90% of cervical smear tests) [17], general practitioners (GP), and midwives. The medical visit and the exam are not free of charge; the letter acts only as a reminder. Women are defined as participating in the organised CCS programme when they have performed a pap smear in the year following a personal invitation for screening following the official governmental guidelines [17]. To increase quality, the management centre organized regular training for health workers performing Pap smears and set up a clinical follow-up in the case of abnormal pap smears.

The VDM screening management centre also managed two types of interventions to reach non-participant women: temporary large-scale CCS information events (for example stall at the market) and interventions in small committees promoting women’s empowerment. These small committee interventions were developed in close collaboration with the local associations working with vulnerable populations.

### Population

In the VDM, the organised CCS programme was initiated in 2010 with an interruption in 2013 and another in 2018. Due to this limitation, we studied the 3 years from January 2014 to December 2016 to compute a screening coverage rate.

From the list of women aged 25–65 years old who live in the VDM, we applied the following exclusion criteria: medical criteria corresponding to women who should not have been invited (death, hysterectomy, history of cervical cancer, disability), women specifically mentioning by mail their refusal to participate, mails returned to sender, or women’s address not geocoded (*n* = 437,022). We then selected women aged from 25 to 63 years old on the first of January 2014, so that each woman remained in the target 25–65 age group during the study period (*n* = 389,205).

As health insurance funding is organised into administrative areas that do not share information, the screening management centre has no visibility regarding CCS and medical history for women who recently moved to the VDM. Therefore, when a woman arrives in the VDM, she is automatically invited to the organised CCS programme. To be sure of women’s CCS history, we thus restricted our analysis to women living in the VDM before the study period (i.e., women having any previous invitation to the CCS programme or cervical pap smear before our study period). We finally restricted our analysis to women who lived in the VDM over the whole study period (i.e., women with a follow-up by the health insurance fund during the whole study period) (*n* = 241,257).

### Variables

For each woman, we collected their address, age, the date of their last cervical pap smear (if any), and the date of the personal invitation to the CCS programme (if any) from the health insurance database. Addresses were geolocalized and assigned to an IRIS (a municipal sub-division including about 2000 people). The age variable was categorized into 10 year-age groups.

For each IRIS (hereafter called neighbourhood), we obtained a social deprivation indicator, the French deprivation index (FDep) [20] using data from the 2013 census. This information was then classified into ten categories according to the IRIS deciles of the VDM distribution as well as the proportion of single women categorized into three categories according to the tertiles of the VDM distribution. When this proportion is low, it could be considered a proxy for living without a partner, an important determinant of participation in CCS [7].

### Analysis

Our outcome was defined as having performed a cervical pap smear during the 3 years 2014–2016. We assessed both relative and absolute inequalities in participation in CCS. Relative inequalities were assessed with an odds ratio. To take into account the hierarchical structure of our data, we conducted a multilevel logistic regression with random effects. We included age as level one and neighbourhood characteristics (proportion of single women and social deprivation) as level two. The CCS coverage rate was calculated by dividing the number of women screened during the 3-year study period by the eligible population. We then measured absolute inequalities using the rate difference by age and neighbourhood characteristics (proportion of single women and social deprivation). We investigated overall screening and opportunistic screening. Women defined as having an opportunistic CCS had performed a cervical pap smear without having received a personal invitation for screening in the year before the test. All statistical analyses were performed with R (3.1 version).

## Results

The characteristics of eligible women aged 25 to 63 and living in the VDM on the first of January 2014 are described in Table 1. The majority of women (55.8%) were over 45 years old. Only 10.3% of women in our sample lived in the two most affluent neighbourhoods (Table1).

**TABLE 1:** Distributions of women aged 25–63 years old living in the Val-de-Marne from January 2014 to January 2017 by age and neighbourghood characteristics (*n* = 241,257) (Paris region, France. 2014–2017).

	N (%)
Individual level	
Age group (years)	
[25–35]	37,407 (15.5)
[35–45]	69,242 (28.7)
[45–55]	75,256 (31.2)
[55–63]	59,352 (24.6)
Neighbourhood level	
Proportion of single women (%) (in tertiles)^a^	
[0–6.2]	80,515 (33.4)
[6.2–10.8]	78,686 (32.6)
[10.8–100]	81,971 (34)
Missing	85
Social deprivation (in deciles)^a^	
Q1 (most deprived)	19,558 (8.1)
Q2	35,359 (14.7)
Q3	29,212 (12.1)
Q4	27,111 (11.2)
Q5	26,280 (10.9)
Q6	29,325 (12.2)
Q7	24,507 (10.2)
Q8	24,688 (10.2)
Q9	18,432 (7.6)
Q10 (least deprived)	6583 (2.7)
Missing	202

a Based on the distribution in the Val-de-Marne.

Tables 2, 3 present social inequalities in CCS participation using absolute and relative measures, respectively. Overall, the CCS coverage rate increased with decreasing age and with decreasing social deprivation whereas it hardly differed by the proportion of single women living in the neighbourhood. Large relative and absolute inequalities were observed for age and social deprivation. For the proportion of single women living in the neighbourhoods, moderate relative inequalities and small absolute inequalities were reported.

**TABLE 2:** Absolute inequalities for participation in cervical cancer screening in the Val-de-Marne from January 2014 to January 2017 by age and neighbourhood characteristics (Paris region, France. 2014–2017).

	Cervical cancer screening coverage^b^		Rate difference		Difference in the coverage between opportunistic and overall screening rate
	Overall screening rate	Opportunistic screening rate^c^	Overall screening rate	Opportunistic screening rate^c^	
Individual level					
Age group (years)					
[25–35]	64.1	51.8	17.5	16.1	−12.3
[35–45]	60.4	48.0	13.8	12.3	−12.0
[45–55]	55.5	45.0	8.9	9.3	−10.5
[55–63]	46.6	35.7	Ref	Ref	−10.9
Neighbourhood level					
Proportion of single women (%) (in tertiles)^a^					
[0–6.2]	55.1	43.4	Ref	Ref	−11.7
[6.2–10.8]	55.3	43.9	0.2	0.5	−11.4
[10.8–100]	57.7	46.6	2.6	3.2	−11.1
Social deprivation (in deciles)^a^					
Q1 (most deprived)	47.1	35.3	Ref	Ref	−11.8
Q2	50.4	38.8	3.3	3.5	−11.6
Q3	52.4	40.7	5.3	5.4	−11.7
Q4	54.7	43.0	7.6	7.7	−11.7
Q5	56.1	44.5	9.0	9.2	−11.6
Q6	59.3	47.9	12.2	12.6	−11.4
Q7	60.5	49.3	13.4	14.0	−11.2
Q8	62.1	51.1	15.0	15.8	−11.0
Q9	62.5	51.6	15.4	16.3	−10.9
Q10 (least deprived)	63.0	52.8	15.9	17.5	−10.2

a Based on the distribution in the Val-de-Marne.

b Calculated over a period of 3 years.

c Women defined as having an opportunistic cervical cancer screening had performed a cervical pap smear without having received a personal invitation for screening in the year before the test.

**TABLE 3:** Relative inequalities for participation in cervical cancer screening in the Val-de-Marne from January 2014 to January 2017 by age and neighbourhood characteristics. Multilevel logistic regression (Paris region, France. 2014–2017).

	Overall screening rate	Opportunistic screening rate^b^
	OR [95% Confidence interval]	OR [95% Confidence Interval]
Individual level		
Age group (years)		
[25–35]	2.13 [2.08–2.19]	2.02 [1.96–2.07]
[35–45]	1.78 [1.74–1.82]	1.69 [1.65–1.73]
[45–55]	1.44 [1.41–1.47]	1.48 [1.45–1.52]
[55–65]	1	1
Neighbourhood level		
Proportion of single women (%) (in tertiles)^a^		
[0–6.2]	1	1
[6.2–10.8]	0.96 [0.93–0.99]	0.97 [0.94–1.00]
[10.8–100]	0.92 [0.88–0.95]	0.93 [0.90–0.96]
Social deprivation (in deciles)^a^		
Q1 (most deprived)	1	1
Q2	1.15 [1.09–1.22]	1.17 [1.11–1.24]
Q3	1.24 [1.17–1.32]	1.27 [1.19–1.35]
Q4	1.38 [1.30–1.47]	1.40 [1.32–1.49]
Q5	1.48 [1.39–1.57]	1.51 [1.42–1.60]
Q6	1.72 [1.61–1.83]	1.76 [1.65–1.87]
Q7	1.82 [1.70–1.94]	1.86 [1.74–1.98]
Q8	1.96 [1.84–2.10]	2.03 [1.90–2.16]
Q9	2.02 [1.88–2.16]	2.10 [1.96–2.24]
Q10 (least deprived)	2.09 [1.90–2.30]	2.22 [2.02–2.44]

a Based on the distribution in the Val-de-Marne.

b Women defined as having an opportunistic cervical cancer screening had performed a cervical pap smear without having received a personal invitation for screening in the year before the test

The CCS rate difference by age was slightly smaller for opportunistic screening compared to overall screening (Table 2). The percentage point difference between the extreme social deprivation groups was slightly higher for opportunistic screening (17.5%) compared to overall screening (15.9%). The CCS rate difference for the proportion of single women living in the neighbourhood hardly differed in both situations.

Using relative measures, the influence of age on participation in screening was higher for overall screening than for opportunistic screening with no overlap in the confidence intervals for the two younger age categories (Table 3). Compared to overall screening, the ORs for the opportunistic screening were almost the same between the two younger age categories, but the OR was higher for the 45–55 year old age category. The ORs for the proportion of single women in the neighbourhoods did not differ between overall and opportunistic screening whereas the social gradient was slightly more pronounced overall than for opportunistic screening.

## Discussion

We investigated disparities in CCS participation by age and neighbourhood characteristics (proportion of single women and social deprivation) in the VDM area that implemented a pilot organised CCS programme. We observed inequalities in CCS participation by age and social deprivation. We found larger relative and absolute inequalities by age but similar or slightly decreased relative and absolute inequalities by social deprivation for overall screening compared to opportunistic screening.

Our analysis is based on a high quality and large population-based sample. Information on CCS participation comes from administrative records and can therefore be considered unbiased. Some deprived groups are nevertheless not included in our database, such as women without health insurance or residence permits. However, we believe this limit does not strongly affect our conclusions due to the very low proportion of this population (<1%). Regarding the transferability of our findings, our results provide insights relevant to other metropolitan areas.

We found larger inequalities regarding age, independent from social deprivation, for overall screening compared to opportunistic screening. Sending invitations increased CCS coverage but our results suggest that it also slightly increased the magnitude of inequalities by age. This stresses the need for a specific strategy targeting older women, who are no longer in their reproductive period and may therefore feel less concerned by gynaecological issues and less likely to maintain regular gynaecological check-ups and smear tests.

In this pilot organised CCS programme, although the test and the medical visit were not free of charge, we observed similar or slightly smaller inequalities by social deprivation for overall screening compared to opportunistic screening. We also found that the absolute increase in CCS rate was higher in women living in the most deprived neighbourhoods. This may be explained by the low opportunistic CCS rate in these groups (rate equal to 35.3% among the women living in the 10% most deprived neighbourhoods), which implies that potentially more women could benefit from the organised CCS programme. However, this may also reflect the effect of the interventions led by the screening management centre that was primarily focused on deprived populations.

We found increased CCS coverage but persisting social inequalities in CCS participation in an area implementing a pilot organised screening programme. This is in line with the literature: the introduction of an organised programme often increased the participation in screening [8, 21, 22] but did not systematically eliminate social inequalities in participation [15, 16, 23, 24]. Several macro-level factors impact these inequalities, including the existence of an organised population-based screening programme, but also the out-of-pocket expenditure, the density of physicians, the implication of health professionals in the promotion and conduction of screening and the level of social protection [23, 25]. The combination of these factors is key for a screening programme to reduce social inequalities in participation. Therefore, tackling the financial barrier to CCS should be a priority in the French pilot screening programme. Indeed, the test and the medical visit are not free of charge. In France, 93% of women have mutual health insurance and they will be fully refunded for the test. Nevertheless, in the VDM as in France, most gynaecologists charge out-of-pocket fees, and most women will have extra costs for the medical visit. The removal of out-of-pocket costs could help further reduce social inequalities in CCS coverage. An intervention in Japan nevertheless observed an increase in inequalities in CCS attendance after removing out-of-pocket fees [16]. This is likely to be partly explained by the small inequalities reported before the intervention [16] and by the well-documented result that interventions initially reach those of higher socioeconomic status and only later target the socially deprived people [26], which could be damaging in term of health inequalities [27]. Finally, in France, healthcare pathways with free CCS exist but they have limited patient capacity and remain unknown to most disadvantaged women. It would be interesting to develop and promote these specific healthcare pathways to increase attendance among disadvantaged women and reduce socioeconomic inequalities.

Other avenues could be suggested to improve the efficiency of the CCS programme in reducing inequalities. The means of communication (by letter) may not be appropriate. Women invited to the organised CCS programme are those who did not have a pap smear within the recommended 3-year interval, these women were thus out of touch with the healthcare system for a long time and a simple invitation for screening by mail may not be enough to re-introduce them in the CCS process. In France, most women have a referring physician and the mention of their name on the invitation for screening could improve the attendance rate [28], in particular among older women and deprived women who are less familiar with the healthcare pathway.

To move forward in the reduction of inequalities, greater involvement of GPs may be considered. In the Netherlands and the UK, GPs are involved in the call and recall process. The UK uses target payments for GPs to encourage them to introduce women to the CCS programme. CCS coverage is approximately 80% in the Netherlands [29] and 70% in the United Kingdom [9]. Even though proving a relationship between this GP involvement, the CCS coverage and the reduction of inequalities remains difficult, this hypothesis could be explored [9]. A CCS strategy through occupational physicians could also be of interest as suggested in China, with free smear tests carried out in state owned enterprises in the textile sector [10]. However, this type of campaign would reach only employed women and this raises the question of equity and equality. Some Eastern European countries such as the Czech Republic combined a pap smear in annual medical examinations in many institutions, with a strong emphasis in the healthcare system on the responsibility of healthcare providers for the timely detection of diseases [8], leading to high CCS rates with low social inequalities even though there is no organised CCS programme [8]. Finally, bringing CCS directly to women either with mobile Pap smear facilities [30] or undertaking urine or vaginal self-sampling [31, 32] could be other options.

It is essential to take social inequalities into account in the implementation of public health programmes to ensure that no one is left behind as well as to monitor and evaluate the effectiveness of approaches. When resources are finite, there may be a tradeoff between maximizing CCS participation while minimizing disparities in screening [33]. Our study provides important results about the burden of social inequalities in participation in CCS and is relevant for French policymakers and public health professionals as a CCS organised programme is currently being implemented at the national level, mimicking the VDM’s organisation. In this national programme, the test is free of charge but not the medical visit. Based on the evidence from countries running organised CCS programmes [23, 25], the pilot screening programme should be improved to reduce social inequalities in participation, and free screening should be proposed. It is also capital that the programme is adequately funded and is actively promoted by well-trained healthcare professionals. Evaluation of the programme following the European guidelines with key performance indicators and cost-effectiveness analyses is required to identify the most sustainable strategies. Moreover, each step of the care pathway from screening to treatment is subject to socio-economic inequalities [34]. The CCS organised programme should therefore also include policies aiming at preventing inequalities in the medical follow-up of pathological results, which could be a challenge for women out-of-touch with the healthcare system.

## Abbreviations

CCS, cervical cancer screening; GP, general practitioner; VDM, Val-de-Marne.
