# Corrigendum: Social Inequalities in Participation in Cervical Cancer Screening in a Metropolitan Area Implementing a Pilot Organised Screening Programme (Paris Region, France)

**DOI:** 10.3389/ijph.2022.1605236

**Published:** 2022-10-05

**Authors:** Céline Audiger, Thomas Bovagnet, Julia Bardes, Gaëlle Abihsera, Jérôme Nicolet, Michel Deghaye, Audrey Bochaton, Gwenn Menvielle

**Affiliations:** ^1^ Le Centre Régional de Coordination des Dépistages des Cancers-CRCDC, Paris, France; ^2^ Sorbonne Université, INSERM, Institut Pierre Louis d’épidémiologie et de Santé Publique (IPLESP UMRS 1136), Paris, France; ^3^ Université Paris Nanterre, UMR CNRS 7533 LADYSS, Nanterre, France

**Keywords:** social inequalities and health inequalities, cervical cancer screening, organised programme, absolute and relative inequalities, Paris area

In the published article, the author’s names were incorrectly written as “Celine Audiger, Gaelle Abihsera, Jerome Nicolet.” The correct spelling’s are “Céline Audiger, Gaëlle Abihsera, Jérôme Nicolet.”

In the published article, there was an error in affiliations **1**, **2**, **3**. Instead of “^1^CRCDC-IDF, Paris, France, ^2^INSERM U1136 Institut Pierre Louis d’Epidémiologie et de Santé Publique, Paris, France, ^3^UMR7533 Laboratoire Dynamiques Sociales et Recomposition des Espaces (LADYSS), Nanterre, France.” It should be “^1^Le Centre Régional de Coordination des Dépistages des Cancers-CRCDC, Paris, France, ^2^Sorbonne Université, INSERM, Institut Pierre Louis d’épidémiologie et de Santé Publique (IPLESP UMRS 1136), Paris, France, ^3^Université Paris Nanterre, UMR CNRS 7533 LADYSS, Nanterre, France.”

In the published article, there was an error regarding the affiliation for Céline Audiger. As well as having affiliation **1**, they should also have affiliation **2**.

The authors apologize for these errors and state that this does not change the scientific conclusions of the article in any way. The original article has been updated.

